# Dissolution-permeation of hot-melt extruded amorphous solid dispersion comprising an experimental grade of HPMCAS

**DOI:** 10.5599/admet.1586

**Published:** 2023-07-22

**Authors:** Hironori Tanaka, Tetsuya Miyano, Hiroshi Ueda

**Affiliations:** 1 Formulation R&D Laboratory, Shionogi & Co., Ltd., Hyogo 660-0813, Japan; 2 Laboratory for Medicinal Chemistry Research, Shionogi & Co., Ltd., Osaka, 561-0825, Japan; 3 Analysis and Evaluation Laboratory, Shionogi & Co., Ltd., Osaka, 561-0825, Japan

**Keywords:** Amorphous, MicroFLUX™, physical stability, Raman mapping, X-ray powder diffraction, extrusion

## Abstract

**Background and purpose:**

Physicochemical properties of an amorphous solid dispersion (ASD) comprising an experimental grade of hydroxypropyl methylcellulose acetate succinate (HPMCAS-MX) with lower glass transition temperature have been previously investigated. This study aimed to evaluate applicability of HPMCAS-MX to hot-melt extrusion (HME) and dissolution-permeation performance of prepared ASDs using MicroFLUX.

**Review approach:**

A physical mixture of indomethacin (IMC) and HPMCAS-MX or -MG (a commercial grade with higher transition temperature) at 20:80 weight ratio was hot-melt extruded to prepare an ASD (IMC-MX and IMC-MG, respectively). The dissolution-permeation performance and the stability of the ASDs were measured.

**Key results:**

A torque reduction at 120 °C implied that IMC-MX transformed into an amorphous state at this temperature, but IMC-MG required around 170 °C. This result was supported by Raman mapping of the the HME samples. IMC-MG and IMC-MX remained in an amorphous state at 40 °C for three months. The initial dissolution rate and solubility of the ASDs were higher than that of crystalline IMC. The apparent permeability of IMC from IMC-MX and IMC-MG was comparable but was approximately two-fold higher than that from crystalline IMC.

**Conclusion:**

HPMCAS-MX enabled HME process at a lower temperature and improved the dissolution-permeation performance of indomethacin.

## Introduction

Oral administration is an important advantage for small molecular medicine because medium and large molecules show lower intestinal membrane permeability, followed by poor oral absorption [1-2]. However, the number of poorly water-soluble compounds has increased and become a key issue for drug discovery. The development of small molecules due to a decrease in aqueous medium solubility could result in reduced absorption per the oral route [3-4]. Solubility issues have been reported in approximately 75 % of new drug candidates. Therefore, physicochemical and pharmaceutical studies to improve solubility are required [3]. Conversion of the drug form to salt and cocrystal and a reduction of the particle size between the micrometer to nanometer range are commonly attempted to modify the physicochemical properties of compounds [4]. Modification of drug properties, and combination with co-solvent, surfactant, pH-modifier, and cyclodextrin, have been considered as potential methods [4]. In addition, amorphization with the disruption of the crystal lattice of the compound has been reported to enhance solubility [4-6]. Since the amorphous form is thermodynamically unstable and might cause crystallization during the formulation process and/or storage, stabilization of amorphous drugs by combination with a hydrophilic polymer as a carrier was designed as polymeric solid dispersion [5,6]. Miscible dispersion of amorphous drugs into a polymeric carrier with the formation of intermolecular interaction could decrease the molecular mobility, which inhibits crystallization of the amorphous drug [7,8].

However, the formulation process is a major concern for the manufacturing of solid dispersion, where solvent-mediated and fusion methods have been utilized [9]. The solvent-mediated technique involves spray-drying drugs and dissolving polymeric carriers in organic and/or aqueous solvents. Rapid evaporation of solvent results in drug amorphization with miscible dispersion into the polymeric carrier at the molecular level. In contrast, hot-melt extrusion is a process that does not use solvent as a representative fusion technique. High-shared mixing of drug and polymeric carrier using a twin rotating screw over the glass transition temperature (*T*_g_) of a polymeric carrier can transform the polymeric carrier to a rubber state. Subsequently, crystalline drug co-melted with rubbery polymeric carrier under high-shared mixing results in a miscible amorphous blend as a solid dispersion. Although both processes are effective, recent environmental concerns have led to hot-melt extrusion being considered more attractive as it is a solvent-free process [8]. Commercial products of solid dispersion prepared by hot-melt extrusion can comprise various polymeric carriers [8]. Polyvinylpyrrolidone, copovidone, and cellulosic polymers are widely used in commercially and in research [8-12]. Studies on polymeric carriers revealed that hydroxypropyl methylcellulose acetate succinate (HPMCAS) has better physical stability and oral absorption than other carriers [13-15] and is used in a commercial product, “Noxafil^>^“.

Although an advantage for solvent-free processes, elevation of the hot-melt extrusion temperature is limited because thermal degradation of the drug and/or polymeric carrier may occur [16]. To overcome this problem, a study on tadalafil, a known heat-sensitive drug with a high melting temperature (*T*_m_), was performed [16]. The addition of surfactant as a plasticizer to a polymeric carrier could cause a reduction in *T*_g_, enabling the hot-melt extrusion process to be performed at a lower temperature [17,18]. Hence, designing and developing a novel solid dispersion carrier with lower *T*_g_ is desirable. In a previous study, we focused on an experimental grade of HPMCAS originally designed with lower *T*_g_ than commercial grade [19]. Physicochemical investigation of its solid dispersion revealed the formation of intermolecular interaction with indomethacin (IMC) inhibited crystallization. Even though the *T*_g_ of an experimental grade HPMCAS was lower than that of commercial grade, their performances were comparable. This study aimed to investigate the applicability of an experimental grade HPMCAS to the hot-melt extrusion process and the dissolution-permeation performance of the extruded solid dispersion. Indomethacin was employed as the model drug, and a commercial grade of HPMCAS was used for comparison.

## Experimental

### Materials

Indomethacin was purchased from Kongo Chemical Co., Ltd. (Toyama, Japan). A commercial grade of HPMCAS “Shin-Etsu AQOAT® type MG” and an experimental grade “type MX” originally developed were kindly gifted by Shin-Etsu Chemical Co., Ltd. (Tokyo, Japan) [19]. GIT-0 lipid (20 wt.% phospholipid dissolved into dodecane), Prisma HT buffer (pH 6.5), and acceptor sink buffer (ASB) were purchased from pION Inc. (MA, USA).

### Preparation of solid dispersion

Solid dispersion formulations were prepared by hot-melt extrusion (HME) process using a micro-conical twin-screw compounder “HAAKE™ mini CTW” (Thermo Fisher Scientific Inc., Tokyo, Japan). Physical mixtures comprising IMC and HPMCAS-MG or HPMCAS-MX at a 20:80 ratio were prepared using a mortar and pestle and 10 g of each physical mixture was subjected into a feeder of the hot-melt extrusion apparatus followed by mixing with twin-screw rotation at 20 rpm. Both carriers showed no thermal degradation up to 250 °C [19], therefore, the preparation temperature was set to be under 250 °C. The HME experiment was performed to confirm the appropriate temperature for the process. Subsequently, the temperature was set at 100 °C, and the mixture was processed for 10 min. The temperature was raised in increments of 10° C from 100 to 170 °C; the HME process was conducted for 10 min at each temperature. The solid dispersion samples prepared at 170 °C were used for the physiochemical and dissolution-permeation studies because the formulations showed amorphization at this temperature. Extruder torque readings were recorded for each temperature after each 10-min process. The hot-melt extruded was milled using a tablet mill JF-001J1 (Takazono Corporation., Tokyo, Japan) at around 15,000 rpm for 1 min. The resulting powders were treated as solid dispersion samples. The sample comprising 20 % IMC and 80 % HPMCAS-MG (or -MX) is termed IMC- MG (or -MX) (20:80) in the current study.

### Scanning electron microscope (SEM)

The hot-melt extruded samples prepared at 170 °C were glued on 12 mm diameter aluminum sample holders using a round carbon seal (Nisshin EM Co., Ltd., Tokyo, Japan). The samples were sputter-coated with an Au alloy using a magnetron sputter (MSP-1S, Vacuum device, Ibaraki, Japan). The shapes and surface morphologies of samples were investigated using an SEM (VE-8800, Keyence Corporation, Osaka, Japan).

### Raman mapping

To measure the amorphization and distribution of IMC into the carrier non-destructively, Raman imaging was performed for the hot-melt extruded samples prepared at 120 and 170 °C. The Raman spectra of crystalline and amorphous IMC were measured using a RAMANtouch laser Raman microscope (Nanophoton Corporation, Osaka, Japan). The samples were placed onto a hole (diameter: 3 mm and depth: 0.2 mm) on an aluminum plate, followed by smoothing of the surface using a spatula. Raman spectroscopic measurement was performed in the carbonyl region between 1800-1500 cm^-1^. The procedure and the conditions of the apparatus were set according to a previous study [19]: excitation wavelength 785.16 nm, excitation power 113.04 mW, ND filter 100 %, spectrograph center wavelength 1400 cm^-1^, grating 600 gr mm^-1^, slit width 50 μm, exposure time 3 s, averaging 1, gain high, readout port low noise, readout speed 2 MHz, CCD temperature -70 °C, and objective lens 10×/NA 0.3. The wavenumber was calibrated using a spectrum of silicon provided by the equipment. An area of the solid dispersions measuring 200×200 μm was evaluated at a resolution of 2000 nm per spot using Raman Imager 2 (Nanophoton Corporation, Osaka, Japan). The Raman image was drawn by the peak ratio of the crystalline and the amorphous IMC; the ranges of 1699.8 to 1700.5 cm^-1^ and 1685.1 to 1695.8 cm^-1^ were used as the peak areas of the crystalline and the amorphous IMC, respectively.

### X-ray powder diffraction

Amorphization of the hot-melt extruded samples was measured by X-ray powder diffraction (XRPD) . The smooth samples were placed on an aluminum plate with holes (diameter = 2 mm, depth = 0.1 mm) and analyzed with a SmartLab (Rigaku Corporation, Tokyo, Japan) instrument equipped with a 9 kW rotating anode using Cu Kα radiation (*λ* = 0.154186 nm) and a HyPix-3000 detector. The distance between the sample and detector was 331 mm; the diffractometer was equipped with a cross-beam optic, providing a parallel beam. A PSC with 2.5° and a slit of 0.05 mm height and 0.5 mm width was used; thus, the beam footprint for all measurement configurations was smaller than that of the sample. No slit was used on the receiving side. The Cu Kα radiation point source was operated at 40 kV and 200 mA. The scan was performed from 2*θ* = 3 to 32°, with *β* axis rotation (20 rpm) during data collection; the sampling step was 2*θ* = 0.02°, and the count time was 40 s. The data were analyzed using Smart Lab studio II X64 version 4.2.111.0 software (Rigaku Corporation, Tokyo, Japan).

### Physical stability

The crystallization tendency of the solid dispersion samples was evaluated. The samples held on a hole (diameter: 3 mm and depth: 0.2 mm) of an aluminum plate were placed into a desiccator with silica gel. The samples in the desiccator were stored at 40 °C for three months, followed by XRPD measurement.

### Dissolution-permeation test

Dissolution and permeation of the crystalline IMC and the solid dispersion samples were simultaneously measured using MicroFLUX™ (pION Inc., MA, USA). The apparatus contains donor and acceptor chambers connected through an artificial membrane (*ϕ* 0.45 μm of polyvinylidenfluoride). The measurement conditions were set by modifying those of previous studies [20]. First, 25 μL of GIT-0 lipid solution was dripped onto the membrane surface from the donor side. Thereafter, 20 mL of PrismaP HT buffer and acceptor sink buffer (ASB)were added to the donor and acceptor chambers. Prisma HT, a pH 6.5 buffer, acts as the donor medium and does not contain phosphate, boric acid, citric acid, or benzoate. ASB is a HEPES-based, pH 7.4 buffer, and acts as the acceptor buffer, which contains chemical scavengers to maintain sink conditions [21,22]. The solid dispersion samples corresponding to 10 mg of IMC were added to the donor chamber. The dissolution media in both chambers were stirred at 150 rpm at 37 °C. The UV probes of 2 and 5 mm were inserted into the donor, and acceptor chambers, respectively, and the concentration of IMC was measured using a UV spectrometer at 370 nm every 5 s (0-10 min), 20 s (10.5-60 min), 1 min (61-120 min), and 2 min (121 – 480 min). The concentration of IMC was corrected with a change in baseline values using the difference between 370 and 380 nm. A standard curve for IMC between 20.0-558.1 μg mL^-1^ in both chambers was plotted. The flux values through the membrane were obtained for crystalline IMC and the solid dispersions of IMC-MG (20 : 80) and IMC-MX (20:80) in accordance with the methods previously described in the literature [23,24]. The flux value was calculated as follows: Flux value, (μg min^-1^) cm^-2^ = the permeated slope, μg mL^-1^) min^-1^ the volume of the acceptor chamber /the surface area of the membrane.

### Solubility test

The solubility of crystalline IMC in the media used in both donor and acceptor chambers of the MicroFLUX™ apparatus was measured. Approximately 50 mg of crystalline IMC was added to 5 mL of Prisma HT (pH 6.5) and ASB (pH 7.4) buffers. They were shaken at 70 cycles/min in an MM-10 water bath shaker (TAITEC, Koshigaya, Saitama, Japan) at 37 °C for 24 h. The test media were filtered through a 0.45-mm syringe filter (GL Sciences Inc., Tokyo, Japan). Approximately 1 mL of the filtrate was analyzed using a Waters ACQUITY UPLC H-class system (Waters Corporation, Massachusetts, USA). The measurement conditions were as follows: mobile phase, 0.1 % trifluoroacetic acid/acetonitrile; flow rate 0.4 mL min^-1^; injection volume 2 μL; UV absorbance 270 nm, and column ACQUITY UPLC BEH C18 2.1 × 100 mm, 1.7μm at 35 °C.

### Measurement of the drug-rich colloid

Prior to the experiment, the PrismaP HT buffer was filtered through a 0.45-mm syringe filter (GL Sciences Inc., Tokyo, Japan). A sample of the solid dispersions (5 mg) corresponding to 1 mg of IMC was added to 2 mL of the filtered PrismaP HT Buffer. They were shaken at 500 rpm at 37 °C for 4 h using a well plate shaker, M BR-022UP (TAITEC Corporation, Saitama, Japan), followed by filtration through a 0.45-μm syringe filter (GL Sciences Inc., Tokyo, Japan). Approximately 100 μL of the filtrate sample was added to a cuvette to measure dynamic light scattering (DLS) using Zetasizer Nano-ZS (Malvern Panalytical Ltd., Malvern, UK). The mean diameter was calculated using photon correlation spectroscopy, and the attenuation and measurement settings were optimized automatically by Zetasizer Software version 8.01 (Malvern Panalytical Ltd., Malvern, UK).

## Results and discussion

### Hot-melt extrusion

**Figure 1.**
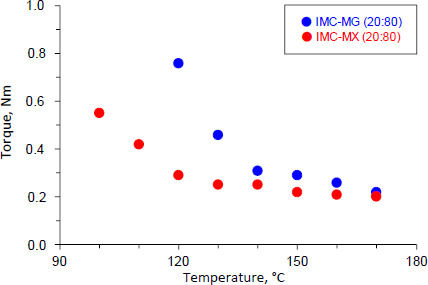
Torque monitoring during hot-melt extrusion of IMC-MG (20:80) and IMC-MX (20:80) at 100-170 °C.

The physical mixture of IMC-MG (20:80) and IMC-MX (20:80) was subjected to the hot-melt extrusion process. Figure 1 shows the torque values as a function of temperature for IMC-MG (20:80) and IMC-MX (20:80). The IMC-MG (20:80) sample was mixed and extruded at 100 °C, but higher torque over 2.5 Nm caused lower processability; the powder could not be fed into the twin-screw barrel. The temperature was elevated to 120 °C, and the process was re-performed. The torque measured 0.76 Nm and subsequently decreased with each temperature elevation, reaching 0.22 Nm at 170 °C. Whereas when the IMC-MX sample was processed at 100 °C, the torque measured approximately 0.55 Nm. The torque was reduced to almost 0.20 Nm at 120 °C. Previously, an HME experiment using a mixture of ibuprofen and Kollidon^>^ SR observed a reduction of torque with processing temperature, and this phenomenon could be correlated with the *T*g of the mixture [25]. The *T*_g_ of HPMC-MG and HPMC-MX were 121.3±1.2 °C and 107.9±0.4 °C, respectively [19]. The observed rapid reduction of torque at temperatures over 120 °C was caused by the mixtures reaching the glass transition temperature of HPMCAS followed by melting of IMC. These results suggest that HPMCAS-MX can be effectively used in the hot-melt extrusion process for the preparation of solid dispersion at a relatively lower temperature than HPMCAS-MG.

**Figure 2.**
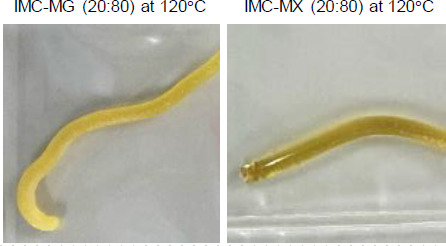
Appearance of hot-melt extruded IMC-MG (20:80) and IMC-MX (20:80) processed at 120 °C.

Figure 2 shows the appearance of the hot-melt extrudate IMC-MG (20:80) and IMC-MX (20:80) processed at 120 °C. The IMC-MG (20:80) extrudate was characterized by a visible dispersion of white powder in the extrudate. In contrast, IMC-MX (20:80) produced a translucent yellow extrudate. The glass transition of IMC results in a change in appearance and a yellow-glassy appearance is formed by the disruption of the crystal lattice due to a change in the form of the intermolecular interaction between functional groups [26-27]. Therefore, it suggests a possibility of partial or complete amorphization with the carrier after hot-melt extrusion. These results were consistent with the change in the torque during the hot-melt extrusion process shown in Figure 1. IMC-MG (20:80) required over 150 °C to reach a plateau in torque reduction, while IMC-MX (20:80) only required 120 °C.

**Figure 3.**
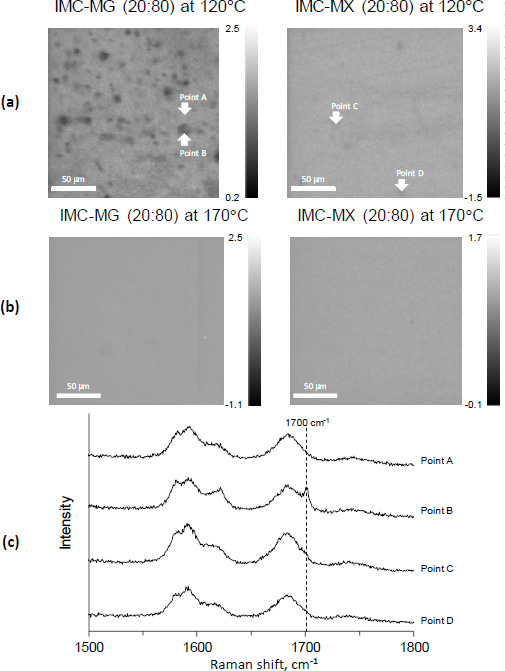
Raman images of IMC-MG (20:80) and IMC-MX (20:80) processed at (a) 120 °C and (b) 170 °C, and Raman spectra of (c) points A-D in the Raman images.

To confirm if the dispersed white powder was crystalline IMC, Raman mapping was performed as a non-destructive method, as the milling process for the preparation of powder to be measured by XRPD might cause amorphization [28]. Figure 3a shows the Raman images of the cutting plane of the hot-melt extrude processed at 120 °C.

The images were drawn using the peak ratio of crystalline and amorphous IMC; the lower intensity is represented as dark gray and reflects crystalline IMC. The distribution image of IMC-MG (20:80) revealed that crystalline IMC remained and was heterogeneously dispersed in the extrude. However, crystalline IMC did not appear in IMC-MX (20:80), and homogenous dispersion of amorphous IMC into the extrude was shown. The results showed that IMC-MX (20:80) could be transformed to solid dispersion by processing at 120 °C, but IMC-MG (20:80) could not. This temperature was lower than the reported values; the study of hot-melt extrusion using HPMCAS set the process temperature above the *T*_g_ at over 130 °C [29,30]. Figure 3b shows the Raman images of the cutting plane of the hot-melt extrude processed at 170 °C. Both the hot-melt extrude of IMC-MG (20:80) and IMC-MX (20:80) processed at 170 °C showed homogeneous distribution without crystalline IMC, suggesting that IMC-MG (20:80) also formed amorphous dispersion.

Figure 3c shows the Raman spectra of points A, B, C, and D, highlighted in Figure 3a. Points B, C, and D highlight the amorphous pattern similar to the previous study [19]. However, the crystalline peak of IMC at 1700 cm^-1^ [31] appeared on point B, corresponding to a dark gray particle in IMC-MG (20:80) at 120 °C. It appears that the Raman image reflects the distribution of crystalline IMC. To compare the performance of the solid dispersion comprising HPMC-MG or HPMC-MX, the solid dispersion prepared at 170 °C under the same conditions were subjected to further study.

### Preparation of the powder and physical stability test

**Figure 4.**
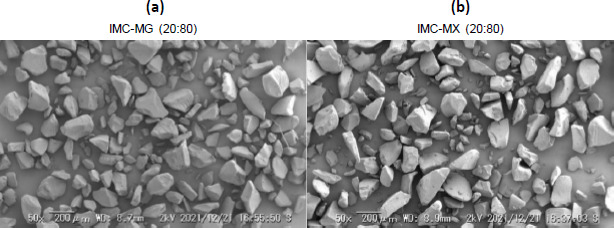
SEM images of hot-melt extruded at 170 °C after milling for (a) IMC-MG (20:80) and (b) IMC-MX (20:80).

The solid dispersion powder was prepared by milling the hot-melt extrude for further experiments. Figure 4a and b show the SEM images of IMC-MG (20:80) and IMC-MX (20:80) prepared by hot-melt extrusion at 170 °C followed by milling. A similar particle shape and size with consolidated and smooth glass-like material can be seen in both images. This appearance was almost consistent with a previous study [32].

**Figure 5.**
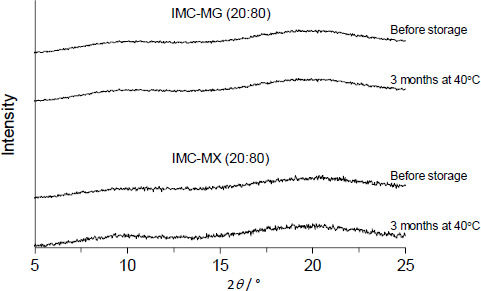
XRPD patterns of hot-melt extruded at 170 °C after milling for IMC-MG (20:80) and IMC-MX (20:80) before and three months after storage at 40 °C.

Figure 5 shows the XRPD patterns of IMC-MG (20:80) and IMC-MX (20:80) prepared by hot-melt extrusion at 170 °C followed by milling. All samples showed no X-ray diffraction peak derived from IMC, suggesting amorphization was achieved following the process. Furthermore, they were physically stable; no crystallization occurred, and the amorphous form was stored for three months at 40 °C. It is commonly known that *T*_g_ is a key parameter used to determine the crystallization tendency of amorphous compounds because the molecular mobility is significantly elevated over *T*_g_ with glass transition [33,34]. In addition, the formation of intermolecular interaction between the drug and polymeric carrier has been reported to play an important role in maintaining an amorphous state [12,15]. Solid dispersion comprising of > 60 % of HPMCAS-MG or HPMCAS-MX showed a *T*_g_ over 70 °C; the formation of intermolecular interaction between IMC and HPMCAS-MG/HPMCAS-MX was characterized by a change in infrared spectra [19]. The difference between the *T*_g_ and storage temperature and the formation of intermolecular interaction with HPMCAS-MG or HPMCAS-MX should result in higher physical stability.

### Dissolution-permeation test

**Figure 6.**
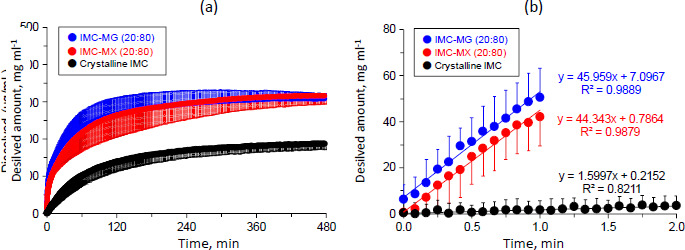
Dissolution profiles of crystalline IMC and solid dispersions of IMC-MG (20:80) and IMC-MX (20:80) in the range of (a) 0-480 min and (b) 0-2.0 min. The error bars represent a standard deviation of n = 3.

Dissolution and the subsequent permeation profiles of crystalline IMC and the solid dispersions were studied with simultaneous measurement for donor-acceptor chambers using a MicroFLUX™. Figure 6a shows the dissolution profiles of crystalline IMC and the solid dispersions of IMC-MG (20:80) and IMC-MX (20:80) prepared by hot-melt extrusion at 170 °C. The concentration of crystalline IMC in the dissolution medium increased time-dependently and almost achieved a plateau at 185.9 ± 0.9 μg mL^-1^ between 420 and 480 min. This result was similar to that of the solubility test; the equilibrium solubility of crystalline IMC for the acceptor medium was 177.2±2.7 μg mL^-1^, which was also similar to the previously reported value (approximately 200 μg mL^-1^) [35]. The dissolution profile of IMC was significantly improved by formulation of the solid dispersion. The initial dissolution rate and dissolved amount of IMC elevated to around 308±0.4 and 316±0.5 μg mL^-1^ between 420-480 min for both IMC-MG (20:80) and IMC-MX (20:80), respectively. Furthermore, no precipitation tendency was observed for both the solid dispersions, keeping supersaturation during the test time. These dissolved amounts of IMC were similar to a previously reported value of a solid dispersion comprising IMC and HPMCAS-LF [36].

Figure 6b shows the magnified dissolution profiles within 2 min. The slope of the concentration as a function of experimental time was calculated between 0-1 min for the solid dispersions, but crystalline IMC was calculated until 2 min because the dissolution was significantly low until 1 min. The initial dissolution rate represented as the slope of crystalline IMC was 1.6 (μg mL^-1^) min^-1^, while the solid dispersion of IMC-MG (20:80) and IMC-MX (20:80) were 46.0 and 44.3 (μg mL^-1^) min^-1^, corresponding to about a 29- and 28-fold bigger improvement, respectively. Improvement of the dissolution rate by the formation of solid dispersion using HPMCAS as a polymeric carrier has been investigated for various drugs [37-39]. The formation of a drug-rich phase of danazol under a supersaturated state was confirmed prior to crystallization, which was affected by the addition of polymeric carriers [37]. In addition, the effect of HPMCAS on the drug-rich phase of atazanavir was studied. The distribution of HPMCAS into a drug-rich phase did not agree between different grades, resulting in dissolution performance [38]. Structural conformation of HPMCAS with random-coil or aggregation affected the supersaturation state of the drug; change in the conformation between grades of HPMCAS impacted the supersaturation of celecoxib [39]. These findings suggest that both grades of HPMCAS used in this study formed similar interactions with IMC in a supersaturated solution, resulting in comparable results for the initial dissolution rate and concentration at a plateau between 420 and 480 min.

**Figure 7.**
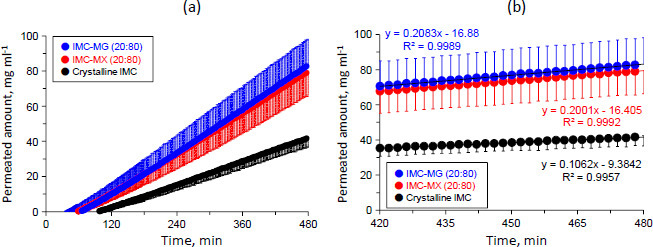
Permeation profiles of crystalline IMC and solid dispersions of IMC-MG (20:80) and IMC-MX (20:80) in the range of (a) 0-480 min and (b) 420-480 min. The error bars represent the standard deviation of n = 3.

Figure 7a shows the permeation profiles of crystalline IMC and the solid dispersions of IMC-MG (20:80) and IMC-MX (20:80). The concentrations of IMC linearly increased depending on experimental time for all samples; both the solid dispersions showed a reduction in lag-time from around 100 to 60-65 min and a higher concentration than that of crystalline IMC. The concentrations of all of the samples were within 100 mg mL^-1^. The solubility test result for crystalline IMC in the acceptor medium was 1.84±0.39 mg mL^-1^. IMC is an acidic drug, and its solubility increase is pH-dependent, especially at pH values greater than 4 [40]. This result suggests that in all samples, IMC was not saturated in the acceptor medium during the dissolution-permeation test. The slope of the samples with concentration and time were calculated in the range of 420 to 480 min, in which the dissolution almost attained a plateau, as described in Figure 7a.

Figure 7b shows the permeation profiles of the samples in the range of 420 to 480 min. The slope of crystalline IMC was 0.11 (μg mL^-1^) min^-1^, while those of the solid dispersion of IMC-MG (20:80) and IMC-MX (20:80) were 0.21 and 0.20 (μg mL^-1^) min^-1^, respectively. The flux values through the membrane were measured for crystalline IMC, and the solid dispersions of IMC-MG (20:80) and IMC-MX (20:80) were measured according to the previously described process [23-24]. The calculated flux values of crystalline IMC and the solid dispersions of IMC-MG (20:80) and IMC-MX (20:80) were 1.38, 2.71, and 2.60 (μg min^-1^) cm^-2^, respectively. Formulation of the solid dispersions resulted in an approximately two-fold increase in the flux value of IMC; no significant differences in the flux values were observed between the two solid dispersions. It is known that the membrane permeability is affected by the formation of drug-rich colloids in the supersaturation state; polymers used as solid dispersion carriers were distributed into drug-rich colloids, which stabilized the drug [36-38]. Resistance of diffusion across the unstirred water layer was reduced by the drug-rich colloids, resulting in enhanced membrane penetration [24]. The sizes of the drug-rich colloids of the solid dispersions in the acceptor buffer were measured; the average sizes were 140.2 and 148.5 nm for the solid dispersion IMC-MG (20:80) and IMC-MX (20:80), respectively, showing no significant difference. Hence, it is suggested that the effects of HPMCAS-MG and HPMCAS-MX on drug-rich colloids are comparable, inducing similar permeation profiles and the flux value of IMC. The results of the dissolution and permeation tests determined that while HPMCAS-MX has a comparable ability to HPMCAS-MG as a solid dispersion carrier, it can undergo the hot-melt extrusion process at a lower temperature.

## Conclusions

This study aimed to evaluate the potential of an experimental grade of HPMCAS-MX for the processability of hot-melt extrusion and improvement of dissolution-permeation of IMC as a solid dispersion carrier compared to a commercial grade of HPMCAS-MG. Monitoring during hot-melt extrusion revealed that torque with high-share mixing was reduced depending on the processing time and temperature. The torque of IMC-MX (20:80) almost attained a plateau at 120 °C, while HPMCAS-MG (20:80) did not. Raman mapping characterized that elevation of the processing temperature up to 170 °C induced transformation to the solid dispersions for both formulations. These results show that the use of HPMCAS-MX, with its lower *T*_g_, enables the hot-melt extrusion process to be conducted at a relatively lower temperature.

The solid dispersions prepared by the hot-melt extrusion process showed no crystallization for three months at 40 °C. The dissolution test, followed by the permeation test by simultaneous measurement of the donor and acceptor chambers, showed significant enhancement of the dissolution rate and dissolved amount of IMC for both the solid dispersions. Moreover, the flux value of IMC in the solid dispersion was higher than that of crystalline IMC, reflecting the improved membrane penetration of the solid dispersions. Therefore, it is concluded that HPMCAS-MX has a comparable ability to the commercial grade product as a solid dispersion carrier for both solid and liquid phases.

This study revealed the applicability of HPMCAS-MX in the hot-melt extrusion process. Further studies using HPMCAS-MX should be conducted to evaluate the effect of the processing temperature on the physicochemical properties of the solid dispersion. In addition, they should investigate the application of our findings to a drug with a high melting temperature.
